# Comparison of the printed and online administration of the Behavioral Regulation in Exercise Questionnaire (BREQ-2)

**DOI:** 10.31744/einstein_journal/2021AO6088

**Published:** 2021-07-23

**Authors:** Nelson Carvas, Igor Conterato Gomes, Juliana Martins Ribeiro Valassi, Luís Anunciação, Ricardo de Freitas-Dias, Marcia Kiyomi Koike

**Affiliations:** 1 Instituto de Assistência Médica ao Servidor Público Estadual “Francisco Morato de Oliveira” São PauloSP Brazil Instituto de Assistência Médica ao Servidor Público Estadual “Francisco Morato de Oliveira”, São Paulo, SP, Brazil.; 2 Universidade de Salvador SalvadorBA Brazil Universidade de Salvador, Salvador, BA, Brazil.; 3 Pontifícia Universidade Católica do Rio de Janeiro Rio de JaneiroRJ Brazil Pontifícia Universidade Católica do Rio de Janeiro, Rio de Janeiro, RJ, Brazil.; 4 Universidade de Pernambuco PetrolinaPE Brazil Universidade de Pernambuco, Petrolina, PE, Brazil.

**Keywords:** Motivation, Motor activity, Exercise, Internet, Online systems, Surveys and questionnaires

## Abstract

**Objective:**

To compare the traditional printed form of the Behavioral Regulation in Exercise Questionnaire with a proposed online form in terms of validity, reliability, and applicability.

**Methods:**

A crossover design study was conducted with 157 undergraduate students. Half of the sample answered the printed questionnaire first and then answered the online questionnaire 7 days later, while the other half of the sample did the inverse. Cronbach's alpha was used to analyze the internal consistency of both the online and printed questionnaires. The construct validity was analyzed by confirmatory factor analysis, using a weighted least square mean and adjusted variance estimation and oblique rotation. The quality of the model was tested with fit indices.

**Results:**

The confirmatory factor analysis showed the 19-item structure with five factors: χ^2^ of 230.718; degrees of freedom of 142; χ^2^/degrees of freedom of 1.625; comparative fit index of 0.978 and root mean square error of approximation of 0.073. All items presented factorial loads above 0.5. There was also excellent consistency between the formats of administration in all dimensions, with Cronbach's alpha values above 0.70. The stability between the formats of administration varied between 0.78 (95%CI: 0.69-0.85) and 0.84 (95%CI: 0.77-0.89), suggesting desirable confidence between both formats of administration.

**Conclusion:**

The five-factor model of the online Behavioral Regulation in Exercise Questionnaire shows internal consistency both in terms of the scale dimensions as well as in terms of the total items.

## INTRODUCTION

Technological advances are changing the way we currently collect data. Research on the reasons for certain people exercising has been traditionally carried out using printed questionnaires.^(^^1^^-^^3^^)^ However, it has become increasingly more common to apply questionnaires through the Internet, *e.g.*, creating an online survey and recruiting participants through social media. Providing an online questionnaire has several advantages in comparison to traditional printed data collection methods. These advantages include reduced costs of administration, faster data gathering, more accurate recording of responses, improved access to participants (especially those in areas physically challenging to reach), improved feeling of anonymity, and reduced ecological impact.^(^^4^^-^^6^^)^

Although the Internet has the potential to be a useful tool to collect information on motivations to exercise, there is no published research on the validity of this kind of online questionnaire. Studies that have systematically reviewed data collection through the Internet, typically focus on reliability^(^^7^^-^^10^^)^ and response rate.^(^^11^^,^^12^^)^ Most of these studies have described small differences between the formats used to administer the questionnaire. Validity is related to quality or condition of the instrument to produce the expected effect.^(^^10^^)^ In this case, the responses obtained by the online instrument must be similar to or close to the values obtained through application in printed format.^(^^11^^,^^12^^)^

However, certain instruments can suffer a significant decrease in validity when adapted to the Internet.^(^^13^^,^^14^^)^ Despite much evidence pointing toward the benefits of administering questionnaires online, levels of validity and reliability are varied and inconsistent, and may have repercussions on the internal and external validity of research. A recent review of several journal databases revealed there is no study published on validity and reliability of the online administration of questionnaires, which have analyzed the motivation regulators for exercising.

Behavioral Regulation in Exercise Questionnaire (BREQ) is a questionnaire that conceptualizes people motivations for physical exercise. While various instruments exist to evaluate the reasons that lead people to exercise, the BREQ^(^^15^^)^ is the most widely used. Behavioral Regulation in Exercise was initially developed to measure the regulation of motives that lead people to exercise, but after receiving criticism (namely for not contemplating items related to amotivation), it was revised to have a second version, entitled BREQ-2.^(^^16^^)^ The BREQ-2 has 19 items that measure the stages of motivation to exercise in five domains (amotivation, external regulation, introjected regulation, identified regulation and intrinsic regulation) on a Likert-type scale, being zero for “not true for me” and four for “very true for me”.

## OBJECTIVE

To compare the traditional printed form of the Behavioral Regulation in Exercise Questionnaire with a proposed online form administered to undergraduate students.

## METHODS

In this crossover study, 158 freshman students from different undergraduate health-related programs were recruited by convenience. To be included in this study, all volunteers were required to be aged over 18 years, and have no medically diagnosed chronic disease, which could hinder physical exercise, and have basic knowledge of computers. Participants who did not fill out the printed questionnaire completely, or those who reported illogical answers on either the printed or online BREQ-2, were excluded from all study analysis.

### Ethics

This study was conducted between March and June 2016 at *Universidade Ibirapuera* (UNIB), as the proposing organization. The Research Ethics Committee of Unib approved this study, with reference number 1.448.110, CAAE: 53855416.8.0000.5597. All participants received information on the study objectives, procedures and the voluntary nature of their participation. Those who accepted to participate signed an informed consent form.

### Behavioral Regulation in Exercise Questionnaire Printed Form

The Brazilian version of the printed BREQ-2 was used for this study.^(^^17^^)^ This questionnaire initiates with the question, “Why do you engage in exercise?”, followed by 19 items to be answered on a Likert-type scale according to the degree that the participant agrees with the statements (zero if “not true for me” to four if “very true for me”).

The responses are grouped into five subscales, according to the self-determination theory (SDT) continuum. The first subscale, “amotivation” corresponds to items 5, 9, 12, and 19; the second subscale, “external regulation” includes items 1, 6, 11, and 16; the third subscale, “introjected regulation”, corresponds to items 2, 7, and 13; the fourth subscale, “identified regulation” includes items 3, 8, 14, and 17; while the fifth subscale, “intrinsic regulation”, corresponds to items 4, 10, 15, and 18.

The BREQ-2 has been used both as a multidimensional instrument that separates the scores for each subscale, and as a unidimensional tool that provides a scale of the degree of self-determination. In this study, the scale was used as a multidimensional instrument, since the analysis was focused on the psychometric properties of the questionnaire. Considering this was a crossover questionnaire-based study, the risk to participants was considered low.

### Online format of Behavioral Regulation in Exercise Questionnaire

The BREQ-2 was adapted to a format to be administered online using the Google Forms. It is a free application for survey administration included in the Google Drive office suite, and has an option to send questionnaires to the participants by e-mail. The BREQ-2 was created to be easy to fill out, with the questions corresponding to each item placed next to multiple-choice selections. In this online version, responding to each question was mandatory, to ensure the responses were submitted only once and all questions were answered. The questionnaire was formatted to fit on one page. After the questionnaire was sent, the data was uploaded automatically into a database, in which each column corresponded to an item, and each line corresponded to a participant.

### Sample size estimate

As previously proposed, to validate psychometric instruments, it is expected to have at least one hundred individuals per assessed factor, or ten to 15 individuals per itens or variables.^(^^18^^)^

### Procedures

The principal investigator of this study contacted participants to invite them to an in-person debriefing session. At the meeting, the investigator explained the objectives of the study and the activities involved, and the volunteers signed a written Informed Consent document. Those who read and signed the Informed Consent form were then assigned a random number, generated by the website Random (www.random.org). This number assigned to each participant to one of two groups was used to match online and printed questionnaires in the database.

### Group I

Participants in Group I received a printed BREQ-2 to be filled out in a classroom during a period defined by their lecturers. After seven days, Group I participants were sought out by the principal investigator and requested to visit the university computer laboratory. At the lab, participants could open the online BREQ-2 using a link previously sent to their email. During the online application in both groups, the computers were at a distance that did not allow the students to consult the answers. The recruiters did not interfere in the responses, they only supervised to prevent side conversations.

### Group II

Group II participants were first sent to the university computer lab, where computers were available for each of them, with a personalized link to the online BREQ-2. Seven days after filling out the online questionnaire, the principal investigator gave participants in Group II a printed BREQ-2 to be filled out in person in a classroom. The seven-day interval between online and printed questionnaires for both groups was used to avoid memory biases, and avoid possible changes in the determinants of behavioral regulation in physical exercise.^(^^9^^)^

### Statistical analysis

The R package, version 3.5.1,^(^^19^^)^ was used for all statistical analysis. The first step was to calculate means, standard deviations, and measurements of asymmetry and kurtosis to explore the assumptions of normality of each item. To have a normal distribution, the values of the indices described should be close to zero, and understood as within an interval ranging from −1.96 to 1.96.^(^^20^^)^

A Cronbach's α coefficient was used to analyze the internal consistency of both the online and printed questionnaires. Values of the Cronbach's α equal to or ≥0.7 indicate that the data are consistent.^(^^21^^,^^22^^)^ The construct validity was analyzed with confirmatory factor analysis (CFA), using a weighted least square mean and variance adjusted (WLSMV) estimator and oblique rotation.^(^^23^^)^ The quality of the model was tested using fit indices: the proportion of χ^2^/degree of freedom (χ^2^/DF), comparative fit index (CFI), root mean square error of approximation (RMSEA) and its respective 95% confidence interval (RMSEA 95%). The proportion of χ^2^/DF is a test of goodness of fit. Values above five correspond to an inadequate fit, values between two and five correspond to an acceptable fit, values between one and two correspond to a close fit, and values below one correspond to a good fit.^(^^20^^)^

The CFI evaluates the adaptation of the theoretical model in comparison with the null model, when the latter is independent from the sample size. Comparative fit index values ≤0.8 indicate a poor adjustment, values between 0.8 and 0.9 indicate an acceptable fit, values between 0.90 and 0.95 indicate a good fit, and values ≥0.95 indicate an excellent fit.^(^^20^^)^ The RMSEA evaluates if the adjusted model is approximately correct compared to the fit obtained, if the minimum discrepancy function was obtained from populational values. Root mean square error of approximation (95% confidence interval – 95%CI: p value: RMSEA ≤0.05) above one suggest an unacceptable adjustment, values between 0.05 and 0.10 suggest an acceptable adjustment, and values of ≤0.05 suggest an excellent adjustment.^(^^20^^)^ The mean variance extracted was calculated to evaluate the proportion of variance of the items, which are explained by the factor to which they belong. A positive convergence of the model is assumed if extracted mean variance values are equal or above 0.5.

To verify the stability of participant response between the two formats, the first step was to calculate the measurements of each subscale of the BREQ-2. The next step was then to compare the rows of each subscale of the online format to the printed format using a non-parametric Wilcoxon-Mann-Whitney test and an intraclass correlation coefficient, to verify the strength of the consistency. A level of significance of 5% was adopted for all analyses.

## RESULTS

### Recruitment and randomization of the study sample

**Figure 1:**
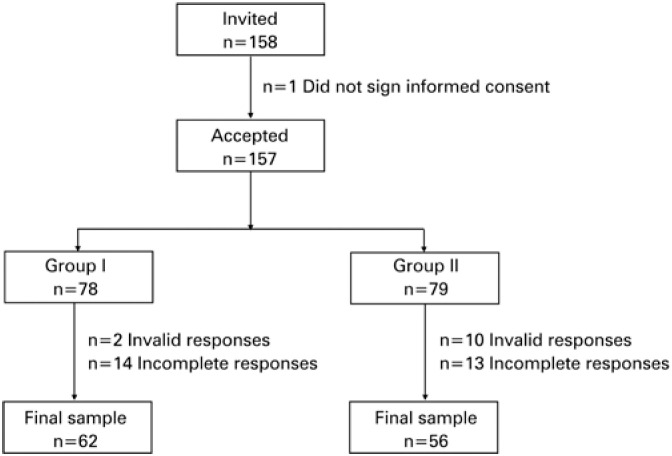
Flowchart of sample distribution

A total of 158 participants were recruited from different undergraduate health sciences courses. One participant refused to sign the informed consent form and was therefore excluded from the study; 12 participants were excluded for straightlining the questionnaire (chosing the same option for all 19 BREQ-2 items); and 27 participants for failure to fill out the questionnaires completely (Figure 1). Thus, the final sample consisted of 118 participants.

### Descriptive analysis of internal consistency of the online Behavioral Regulation in Exercise Questionnaire

Means and standard deviations, along with indices of asymmetry and kurtosis, and the Cronbach's α of each item obtained on the online BREQ-2 are presented in table 1. Scores obtained on the items corresponding to external regulation and amotivation were not normally distributed; asymmetry and kurtosis were above the 1.96 intervals. The highest indices of asymmetry and kurtosis were obtained on item 11 (“I exercise because others will not be pleased with me if I don't”), corresponding to external regulation, and item 19 (“I think exercising is a waste of time”), corresponding to amotivation. Mean values varied between 0.24 to 0.67 and 3.06 to 1.18. The Cronbach's alpha, for these same items, was found to vary from 0.67 (external regulation) to 0,82 (intrinsic regulation), while the variation between all items ranged between 0.80 and 0.85. These findings suggest that the online administration of the BREQ-2 is associated with desirable indices of internal consistency.

**Table 1:** Results of the Behavioral Regulation in Exercise Questionnaire administered to undergraduate students

Online		Mean	Standard deviation	Asymmetry	Kurtosis	Cronbach's alpha
Amotivation						α=0.74
	Item 5	0.41	0.95	2.34	4.64	0.85
	Item 9	0.50	1.03	2.16	3.83	0.82
	Item 12	0.57	1.12	1.89	2.34	0.82
	Item 19	0.24	0.67	3.28	11.47	0.82
External regulation						α=0.67
	Item 1	0.46	0.99	2.22	4.18	0.82
	Item 6	0.32	0.72	2.6	7.23	0.81
	Item 11	0.27	0.73	3.14	9.88	0.81
	Item 16	0.45	0.92	2.11	3.69	0.82
Introjected regulation						α=0.72
	Item 2	1.66	1.42	0.29	−1.2	0.80
	Item 7	0.83	1.2	1.25	0.43	0.80
	Item 13	1.50	1.5	0.54	−1.16	0.80
Identified regulation						α=0.71
	Item 3	3.06	1.18	−0.94	−0.38	0.80
	Item 8	2.69	1.25	−0.49	−0.95	0.80
	Item 14	2.77	1.18	−0.53	−0.85	0.81
	Item 17	1.21	1.40	0.74	−0.9	0.80
Intrinsic regulation						α=0.82
	Item 4	1.60	1.46	0.4	−1.25	0.81
	Item 10	2.13	1.41	−0.2	−1.23	0.80
	Item 15	2.64	1.25	−0.44	−1.01	0.80
	Item 18	2.59	1.43	−0.57	−1.07	0.80

n=118.

### Factorial structure of the online Behavioral Regulation in Exercise Questionnaire

A CFA sustained the hypothesis of presence of all five dimensions in the online BREQ-2. This was demonstrated through the equivalent fit indices: χ^2^=230,718; DF=142; χ^2^/DF=1.625; CFI=0.978 and RMSEA=0.073. The load factors oscillated between 0.593 and 0.882, which allowed presuming the online BREQ-2 had construct validity (Table 2).

**Table 2:** Confirmatory factor analysis of the online Behavioral Regulation in Exercise Questionnaire administered to undergraduate students

Online BREQ-2	Amotivation	External regulation	Introjected regulation	Identified regulation	Intrinsic regulation
Item 5	0.786				
Item 9	0.716				
Item 12	0.862				
Item 19	0.769				
Item 1		0.679			
Item 6		0.847			
Item 11		0.822			
Item 16		0.756			
Item 2			0.685		
Item 7			0.83		
Item 13			0.757		
Item 3				0.850	
Item 8				0.774	
Item 14				0.593	
Item 17				0.697	
Item 4					0.635
Item 10					0.881
Item 15					0.747
Item 18					0.882
Average variance extracted	0.617	0.462	0.469	0.722	0.404
Total variance	0.38				

BREQ-2: Behavioral Regulation in Exercise Questionnaire.

n=118.

The values associated with the average variance extracted of the model factors varied from 0.469 (external regulation) to 0.722 (identified regulation). The items that most discriminated each factor were item 12 for amotivation (“I don't see the point in exercising”); item 6 for external regulation (“I take part in exercise because my friends/family/partner say I should”); item 7 for introjected regulation, (“I feel ashamed when I miss an exercise session”); item 3 for identified regulation (“I value the benefits of exercise”); and item 18 for intrinsic regulation (“I get pleasure and satisfaction from participating in exercise”).

### Stability of dimensions in the formats of the Behavioral Regulation in Exercise Questionnaire

Table 3 presents mean values with respective standard deviations for both traditional printed and online format of administration of BREQ-2. The Mann-Whitney U test revealed there was no significant difference in the dimensions of the BREQ-2 between the formats. There was also excellent consistency between the formats of administration in all dimensions, with intraclass correlation coefficient values between 0.78 (95%CI: 0.69-0.85) and 0.84 (95%CI: 0.77- 0.89), suggesting desirable confidence between both formats of administration (Table 4).

**Table 3:** Profiles of association between the dimensions of the online Behavioral Regulation in Exercise Questionnaire

	Amotivation	External regulation	Introjected regulation	Identified regulation	Intrinsic regulation
Amotivation	1.00				
External regulation	0.74	1.00			
Introjected regulation	0.15	0.45	1.00		
Identified regulation	−0.15	0.06	0.73	1.00	
Intrinsic regulation	−0.34	−0.01	0.50	0.80	1.00

**Table 4:** Comparison and consistency between the dimensions of the Behavioral Regulation in Exercise Questionnaire

BREQ-2 dimensions	Printed	*Online*	p value*	ICC (95%CI)
	Mean±SD	Mean±SD		
Amotivation	0.51±0.79	0.43±0.72	0.170	0.83 (0.76-0.88)
External regulation	0.48±0.64	0.38±0.60	0.480	0.80 (0.71-0.86)
Introjected regulation	1.56±1.05	1.33±1.10	1.000	0.78 (0.69-0.85)
Identified regulation	2.59±0.81	2.43±0.92	0.120	0.79 (0.70-0.85)
Intrinsic regulation	2.45±1.05	2.24±1.12	0.100	0.84 (0.77-0.89)

* Mann-Whitney test.

BREQ-2: Behavioral Regulation in Exercise Questionnaire; SD: standard deviation; ICC: intraclass correlation coefficient; 95%CI: confidence interval 95%.

## DISCUSSION

The present study evaluated the validity and consistency of the BREQ-2 and provided evidence of its clinical applicability in an online context. The five-factor model of the online BREQ-2 showed internal consistency, both in terms of the scale dimensions as well as total items. In fact, all extracted behavioral regulators presented internal consistency with a Cronbach's α above 0.7 (except external regulation, with an alpha of 0.67).

One of the possible explanations could lie in the theory behind external regulation, stating people perform actions to satisfy the expectations of those around them. External regulation can also be interpreted as a feeling of guilt when missing an exercise session, which makes this factor similar to introjected regulation.^(^^24^^)^ However, considering the factor associated with external regulation presents a higher number of items compared with introjected regulation, it is assumed the internal consistency could be marginal.^(^^20^^)^ These findings suggest the online administration of the BREQ-2 is associated with desirable indices of internal consistency.

The findings of this study are similar to those found in other versions of the BREQ-2, which were translated into other languages. Regarding the factorial structure of the online format of the BREQ-2, the results present satisfactory levels of fit, with a similar factorial arrangement found in both the original study^(^^15^^,^^16^^)^ and in the other language versions.^(^^17^^,^^25^^-^^30^^)^ Measuring the direction of the associations between the dimensions offers another analytical method to analyze the validity of the theoretical factorial structure underlying the online BREQ-2. These associations should move in the same direction as the responses on the SDT continuum. In this case, the covariance between the dimensions presented, confirms the presence of a self-determination theory continuum, since the regulations close to each other in the continuum were shown to be strongly correlated in a positive direction, when compared with regulations further away in the continuum.

Several studies have shown that adapting questionnaires and other data collection instruments to the Internet does not compromise their measurements.^(^^7^^-^^9^^,^^31^^)^ Reports of more positive results of online instruments when compared with traditional printed tools have also been described in the literature.^(^^9^^)^ It is hypothesized that administering questionnaires over the Internet reduces the tendency to provide socially desirable answers. This is because computers could provide a more impersonal situation, in which individuals feel more anonymous and less concerned about how they may be perceived by other people.^(^^5^^,^^9^^)^

No differences were found between the two different formats to administer the BREQ-2 to undergraduate students. Elevated intraclass correlation coefficient values were observed, indicating excellent stability in the responses obtained through the online format of the BREQ-2 in comparison with the traditional printed format. There were slight differences between the formats in terms of the factorial structure, regarding the adjustment of the model to data for online administration.

Despite the promising findings, this study still presented several limitations, which should be detailed. The first limitation, as with many questionnaires, is that all answers are self-reported; this means that answers may not necessarily correspond with the participants’ actual physical activity. However, self-report is a common procedure for data collection using questionnaires and other instruments, since it is the most viable method to collect data on perceptions and beliefs. The fact that certain online items presented slight deviations from a normal distribution should also be remembered. However, in this study, the CFA was carried out with the WLSMV method of estimation with oblique rotation, which contemplates the underlying theory and assumes the data does not have a multivariate normal distribution.^(^^23^^)^ Another limitation of this study consists of the fact that, while the sample size was relatively large in comparison with the sample universe, the selection of participants was not made at random. This lack of random selection could mean the participants were not truly representative of the wider population.

Moreover, we did not investigate the socioeconomic aspects of the participants, which prevented us from evaluating the relation between the responses and these characteristics of the sample, and generalizing our results. Nonetheless, once the instrument is valid for online application, studies evaluating the relation among various aspects, including sociodemographic characteristics, may be carried out.

## CONCLUSION

The responses obtained by the administration of the online format of the Behavioral Regulation in Exercise Questionnaire were not statistically different from the values obtained with the responses gathered in the printed format. Furthermore, desirable values were observed in psychometric performance, in relation to the factorial solution and measures of reliability of the responses obtained by the online administration of the instrument.
