# Supplementary material for: Comparison of the printed and online administration of the Behavioral Regulation in Exercise Questionnaire (BREQ-2)
Source: Einstein (Sao Paulo). 2021 Jul 23;19:eAO6088. doi: 10.31744/einstein_journal/2021AO6088 (PMC8302226; doi:10.31744/einstein_journal/2021AO6088)
Supplement: Translation

# Translation: Comparação da administração impressa e *on-line* do *Behavioral Regulation in Exercise Questionnaire* (BREQ-2)

**Authors:** Nelson Carvas, Igor Conterato Gomes, Juliana Martins Ribeiro Valassi, Luís Anunciação, Ricardo de Freitas-Dias, Marcia Kiyomi Koike

**Affiliations:** 1 São PauloSP Brasil Instituto de Assistência Médica ao Servidor Público Estadual “Francisco Morato de Oliveira”, São Paulo, SP, Brasil.; 2 SalvadorBA Brasil Universidade de Salvador, Salvador, BA, Brasil.; 3 Rio de JaneiroRJ Brasil Pontifícia Universidade Católica do Rio de Janeiro, Rio de Janeiro, RJ, Brasil.; 4 PetrolinaPE Brasil Universidade de Pernambuco, Petrolina, PE, Brasil.

**Keywords:** Motivação, Atividade motora, Exercício, Internet, Sistemas on-line, Inquéritos e questionários

## Abstract

**Objetivo:**

Comparar a forma tradicional impressa *do Behavioral Regulation in Exercise Questionnaire* com uma proposta de formulário *on-line*, em termos de validade, confiabilidade e aplicabilidade.

**Métodos:**

Estudo de delineamento cruzado (*crossover*) realizado com 157 estudantes universitários de graduação. Metade da amostra respondeu primeiro ao questionário impresso e, 7 dias depois, ao questionário *on-line*, enquanto a outra metade da amostra fez o inverso. O coeficiente alfa de Cronbach foi usado para analisar a consistência interna dos questionários *on-line* e impressos. A validade de construção foi verificada por análise fatorial confirmatória, utilizando-se um estimador de mínimos quadrados ajustados pela média e variância e rotação oblíqua. A qualidade do modelo foi testada com índices de ajuste.

**Resultados:**

A análise fatorial confirmatória mostrou a estrutura de 19 itens com cinco fatores: χ^2^ de 230,718; graus de liberdade de 142; χ^2^/grau de liberdade de 1,625; índice de ajuste comparativo de 0,978 e raiz do erro quadrático médio de aproximação de 0,073. Todos os itens apresentaram cargas fatoriais acima de 0,5. Também houve excelente consistência entre os formatos de administração em todas as dimensões, com valores de alfa de Cronbach acima de 0,70. A estabilidade entre os formatos de administração variou entre 0,78 (IC95%: 0,69-0,85) e 0,84 (IC95%: 0,77-0,89), sugerindo confiança desejável entre os dois formatos de administração.

**Conclusão:**

O modelo de cinco fatores do *Behavioral Regulation in Exercise Questionnaire on-line* apresenta consistência interna tanto em relação às dimensões da escala quanto em relação ao total de itens.

## INTRODUÇÃO

Os avanços tecnológicos mudam conforme os dados são coletados atualmente. A pesquisa sobre as razões para certas pessoas praticarem exercícios físicos é tradicionalmente realizada utilizando-se questionários impressos.^(1-3)^ No entanto, é cada vez mais frequente aplicar questionários por meio da Internet, por exemplo, criando uma pesquisa *on-line* e recrutando participantes em redes sociais. Administrar um questionário *on-line* tem diversas vantagens em comparação aos métodos tradicionais de coleta de dados em formato impresso. Tais vantagens incluem redução nos custos de administração, coleta mais rápida de dados, registro mais preciso das respostas, melhor acesso aos participantes (especialmente aqueles em áreas de difícil acesso físico), maior sensação de anonimato e impacto ecológico reduzido.^(4-6)^

Apesar de a Internet ter potencial para ser uma ferramenta útil para coletar informações sobre motivações para se exercitar, não há nenhuma pesquisa publicada sobre a validade desse tipo de questionário *on-line*. Estudos que revisaram sistematicamente a coleta de dados por meio da Internet em geral abordam confiabilidade^(7-10)^ e taxa de resposta.^(11,12)^ A maioria deles descreve pequenas diferenças entre os formatos usados para administrar o questionário. A validade está relacionada à qualidade ou à condição do instrumento para produzir o efeito esperado.^(10)^ Neste caso, as respostas obtidas pelo instrumento *on-line* devem ser semelhantes ou próximas aos valores obtidos de sua aplicação em formato impresso.^(11,12)^

Entretanto, certos instrumentos podem sofrer diminuição significativa na validade quando adaptados à Internet.^(13,14)^ Apesar das diversas evidências que apontam para os benefícios da aplicação de questionários *on-line*, os níveis de validade e confiabilidade são variados e inconsistentes, o que pode ter consequências na validade interna e externa da pesquisa. Uma busca recente nas bases de dados mostrou que não há estudo publicado sobre a validade e a confiabilidade da administração *on-line* de questionários, que avaliaram os reguladores de motivação da prática de exercício físico.

O *Behavioral Regulation in Exercise Questionnaire* (BREQ) conceitua as motivações das pessoas para o exercício físico. Embora existam vários instrumentos para avaliar as razões que levam as pessoas a fazer exercício, o BREQ^(15)^ é o mais utilizado. Ele foi inicialmente desenvolvido para medir a regulação dos motivos que levam as pessoas a exercitar, mas, após receber críticas por não contemplar itens relacionados à amotivação, foi revisado para ter a segunda versão, chamada BREQ-2.^(16)^ O BREQ-2 tem 19 itens que medem os estágios da motivação para se exercitar em cinco domínios (amotivação, regulação externa, regulação introjetada, regulação identificada e regulação integrada) em uma escala do tipo Likert, na qual zero significa “nada verdadeiro para mim” e quatro, “muito verdadeiro para mim”.

## OBJETIVO

Comparar a forma tradicional impressa de *Behavioral Regulation in Exercise Questionnaire* com um formulário *on-line* administrado a estudantes de graduação.

## MÉTODOS

Neste estudo de delineamento cruzado (*crossover*), 158 estudantes do primeiro ano de graduação de diferentes programas relacionados à saúde foram recrutados por conveniência. Para serem incluídos neste estudo, todos os voluntários tinham que ter mais de 18 anos, não ter doença crônica diagnosticada clinicamente, que pudesse impedir o exercício físico e possuir conhecimentos básicos de informática. Os participantes que não preencheram completamente o questionário impresso ou que relataram respostas ilógicas no BREQ-2 impresso ou *on-line* foram excluídos de todas as análises do estudo.

### Ética

Este estudo foi realizado entre março e junho de 2016, na Universidade Ibirapuera (UNIB) como instituição proponente. O Comitê de Ética em Pesquisa da Unib aprovou este estudo, com o número de referência 1.448.110 e CAAE: 53855416.8.0000.5597. Todos os participantes receberam informações sobre os objetivos do estudo, os procedimentos e a natureza voluntária de sua participação. Aqueles que aceitaram participar assinaram um Termo de Consentimento Livre e Esclarecido.

### Formulário impresso do *Behavioral Regulation in Exercise Questionnaire*

A versão brasileira do BREQ-2 impressa foi utilizada para este estudo.^(17)^ Este questionário começa com a pergunta: “Por que você se exercita?”, seguida por 19 itens a serem respondidos em uma escala do tipo Likert, de acordo com o grau de concordância do participante com as afirmações (zero para “nada verdadeiro para mim” a quatro para “muito verdadeiro para mim”).

As respostas estão agrupadas em cinco subescalas, de acordo com o *continuum* da teoria da autodeterminação (TAD). A primeira subescala, “amotivação”, corresponde aos itens 5, 9, 12 e 19; a segunda subescala, “regulação externa”, inclui os itens 1, 6, 11 e 16; a terceira subescala, “regulação introjetada”, corresponde aos itens 2, 7 e 13; a quarta subescala, “regulação identificada”, inclui os itens 3, 8, 14 e 17; e a quinta subescala, “regulação integrada”, corresponde aos itens 4, 10, 15 e 18.

O BREQ-2 tem sido usado tanto como um instrumento multidimensional que separa as pontuações para cada subescala, quanto como ferramenta unidimensional, que fornece uma escala do grau de autodeterminação. Neste estudo, a escala foi usada como instrumento multidimensional, pois a análise foi focada nas propriedades psicométricas do questionário. Como este estudo teve delineamento cruzado e foi baseado em questionário, o risco para os participantes foi considerado baixo.

### Formato *on-line* do *Behavioral Regulation in Exercise Questionnaire*

O BREQ-2 foi adaptado para um formato a ser administrado *on-line* usando o *Google Forms*, que é um aplicativo gratuito de administração de pesquisas incluído no pacote do *Google Drive* e tem uma opção para enviar questionários para os participantes por *e-mail*. Esse questionário foi criado para ser fácil de preencher, com as perguntas correspondentes a cada item colocadas ao lado de seleções de múltipla escolha. Nesta versão *on-line*, a resposta a cada pergunta foi obrigatória, para garantir que as respostas fossem enviadas somente uma vez, e todas as perguntas fossem respondidas. O questionário foi formatado para caber em uma página. Após seu envio, os dados foram carregados automaticamente em um banco de dados, no qual cada coluna correspondia a um item, e cada linha correspondia a um participante.

### Estimativa do tamanho da amostra

Como já proposto, para validar instrumentos psicométricos, espera-se ter, pelo menos, cem indivíduos por fator avaliado, ou dez a 15 indivíduos por itens ou variáveis.^(18)^

### Procedimentos

O investigador principal deste estudo entrou em contato com os participantes, a fim de convidá-los para uma sessão de informação presencial. Na reunião, o investigador explicou os objetivos do estudo e as atividades envolvidas, e os voluntários assinaram um documento de Consentimento Livre e Esclarecido por escrito. Aos que leram e assinaram o Termo de Consentimento Livre e Esclarecido foi, então, atribuído um número aleatório, gerado pelo *site Random* (www.random.org). O número atribuído a cada participante para um dos dois grupos foi usado para fazer correspondência entre os questionários *on-line* e impressos no banco de dados.

### Grupo I

Os participantes do Grupo I receberam um questionário impresso BREQ-2 a ser preenchido em uma sala de aula, durante um período definido por seus professores. Após 7 dias, os participantes do Grupo I foram procurados pelo investigador principal e solicitados a visitar o laboratório de informática da universidade. No laboratório, os participantes puderam abrir o BREQ-2 *on-line* usando um *link* previamente enviado a seu *e-mail*. Durante a inscrição *on-line* em ambos os grupos, os computadores estavam a uma distância que não permitia que os estudantes consultassem as respostas uns dos outros. Os recrutadores não interferiram nas respostas e apenas supervisionaram para evitar conversas entre os participantes.

### Grupo II

Os participantes do Grupo II foram enviados primeiramente ao laboratório de informática da universidade, onde os computadores estavam disponíveis para cada um deles, com um *link* personalizado para o BREQ-2 *on-line*. Sete dias após o preenchimento do questionário *on-line*, o investigador principal deu aos participantes do Grupo II um questionário impresso do BREQ-2 para ser preenchido pessoalmente em uma sala de aula. O intervalo de 7 dias entre questionários *on-line* e impresso para ambos os grupos foi usado para evitar vieses de memória e possíveis mudanças nos determinantes da regulação comportamental nos exercícios.^(9)^

### Análise estatística

O pacote do programa R, versão 3.5.1,^(19)^ foi usado para todas as análises estatísticas. O primeiro passo foi calcular médias, desvios-padrão e medidas de assimetria e curtose, para explorar as suposições de normalidade de cada item. Para ter distribuição normal, os valores dos índices descritos deveriam ser próximos de zero e entendidos como dentro de um intervalo que varia de −1,96 a 1,96.^(20)^

O coeficiente alfa de Cronbach foi usado para analisar a consistência interna dos questionários *on-line* e impressos. Valores do alfa de Cronbach iguais ou ≥0,7 indicam que os dados são consistentes.^(21,22)^ A validade da construção foi analisada com a análise fatorial confirmatória (AFC), utilizando estimador de mínimos quadrados ajustados por média e variância (WLSMV) e rotação oblíqua.^(23)^ A qualidade do modelo foi testada utilizando índices de ajuste: a proporção de χ^2^/grau de liberdade (χ^2^/GL), índice de ajuste comparativo (CFI), raiz do erro quadrático médio de aproximação (REQMA) e seu respectivo intervalo de confiança de 95% (REQMA 95%). A proporção de χ^2^/GL é um teste de grau de adequação. Valores acima de cinco correspondem a um ajuste inadequado, valores entre dois e cinco correspondem a um ajuste aceitável, valores entre um e dois correspondem a um ajuste próximo, e valores abaixo de um correspondem a um bom ajuste.^(20)^

O CFI avalia a adaptação do modelo teórico em comparação com o nulo, quando este é independente do tamanho da amostra. Os valores de CFI ≤0,8 indicam ajuste ruim, valores entre 0,8 e 0,9 indicam ajuste aceitável, valores entre 0,90 e 0,95 indicam bom ajuste, e valores ≥0,95 indicam ajuste excelente.^(20)^ O REQMA avalia se o modelo ajustado é aproximadamente correto em comparação com o obtido, caso a função de discrepância mínima tenha sido obtida a partir de valores populacionais. O erro quadrático médio de aproximação (intervalo de confiança de 95% – IC95% – valor de p: REQMA ≤0,05) acima de um sugere ajuste inaceitável, valores entre 0,05 e 0,10 sugerem ajuste aceitável, e valores de ≤0,05 sugerem ajuste excelente.^(20)^ A variância média extraída foi calculada para avaliar a proporção de variância dos itens, que são explicados pelo fator a que pertencem. Uma convergência positiva do modelo é presumida se os valores da variância média extraída forem iguais a ou acima de 0,5.

Para verificar a estabilidade da resposta dos participantes entre os dois formatos, o primeiro passo foi calcular as medidas de cada subescala no BREQ-2. O passo seguinte foi comparar as colunas de cada subescala do formato *on-line* com o formato impresso, por meio do teste não paramétrico de Wilcoxon-Mann-Whitney e um coeficiente de correlação intraclasse, para verificar a força da consistência. Um nível de significância de 5% foi adotado para todas as análises.

## RESULTADOS

### Recrutamento e randomização da amostra do estudo

Foram recrutados 158 participantes de diferentes cursos de graduação na área de ciências da saúde. Um participante recusou-se a assinar o Termo de Consentimento Livre e Esclarecido e foi excluído do estudo; 12 participantes foram excluídos por terem uniformizado as respostas (escolheram a mesma alternativa para todos os 19 itens do BREQ-2) e 27 participantes por não terem preenchido completamente os questionários (Figura 1). Assim, a amostra final teve 118 participantes.

**Figura 1:** Fluxograma da distribuição da amostra

### Análise descritiva de consistência interna da versão *on-line* do *Behavioral Regulation in Exercise Questionnaire*

As médias e os desvios-padrão, juntamente dos índices de assimetria e curtose, e o alfa de Cronbach de cada item obtido no BREQ-2 *on-line* são apresentados na tabela 1. As notas obtidas nos itens correspondentes à regulação externa e amotivação não apresentaram distribuição normal; a assimetria e a curtose estavam acima dos intervalos de 1,96. Os índices mais altos de assimetria e curtose foram obtidos nos itens 11 (“Faço exercício porque os outros vão ficar insatisfeitos comigo se não fizer”), correspondendo à regulação externa, e 19 (“Penso que o exercício é uma perda de tempo”), correspondendo a uma motivação. Os valores médios variaram entre 0,24 a 0,67 e 3,06 a 1,18. O alfa de Cronbach, para esses mesmos itens, variou de 0,67 (regulação externa) a 0,82 (regulação integrada), enquanto a variação entre todos os itens variou de 0,80 a 0,85. Esses achados sugerem que a administração *on-line* do BREQ-2 esteja associada a índices desejáveis de consistência interna.

**Tabela 1:** Resultados do *Behavioral Regulation in Exercise Questionnaire* administrado a estudantes de graduação

*On-line*		Média	Desvio-padrão	Assimetria	Curtose	Alfa de Cronbach
Amotivação						α=0,74
	Item 5	0,41	0,95	2,34	4,64	0,85
	Item 9	0,50	1,03	2,16	3,83	0,82
	Item 12	0,57	1,12	1,89	2,34	0,82
	Item 19	0,24	0,67	3,28	11,47	0,82
Regulação externa						α=0,67
	Item 1	0,46	0,99	2,22	4,18	0,82
	Item 6	0,32	0,72	2,6	7,23	0,81
	Item 11	0,27	0,73	3,14	9,88	0,81
	Item 16	0,45	0,92	2,11	3,69	0,82
Regulação introjetada						α=0,72
	Item 2	1,66	1,42	0,29	−1,2	0,80
	Item 7	0,83	1,2	1,25	0,43	0,80
	Item 13	1,50	1,5	0,54	−1,16	0,80
Regulação identificada						α=0,71
	Item 3	3,06	1,18	−0,94	−0,38	0,80
	Item 8	2,69	1,25	−0,49	−0,95	0,80
	Item 14	2,77	1,18	−0,53	−0,85	0,81
	Item 17	1,21	1,40	0,74	−0,9	0,80
Regulação integrada						α=0,82
	Item 4	1,60	1,46	0,4	−1,25	0,81
	Item 10	2,13	1,41	−0,2	−1,23	0,80
	Item 15	2,64	1,25	−0,44	−1,01	0,80
	Item 18	2,59	1,43	−0,57	−1,07	0,80

n=118.

### Estrutura fatorial da versão *on-line* do *Behavioral Regulation in Exercise Questionnaire*

Uma AFC sustentou a hipótese da presença de todas as cinco dimensões na versão *on-line* do BREQ-2. Isso foi demonstrado por meio dos índices de ajuste equivalentes: χ^2^=230.718; GL=142; χ^2^/GL=1,625; CFI=0,978 e REQMA=0,073. Os fatores de carga oscilaram entre 0,593 e 0,882, o que permitiu supor que o BREQ-2 *on-line* teve validade de construção (Tabela 2).

**Tabela 2:** Análise fatorial confirmatória da versão *on-line* do *Behavioral Regulation in Exercise Questionnaire* administrado a estudantes de graduação

BREQ-2 *on-line*	Uma motivação	Regulação externa	Regulação introjetada	Regulação identificada	Regulação integrada
Item 5	0,786				
Item 9	0,716				
Item 12	0,862				
Item 19	0,769				
Item 1		0,679			
Item 6		0,847			
Item 11		0,822			
Item 16		0,756			
Item 2			0,685		
Item 7			0,83		
Item 13			0,757		
Item 3				0,850	
Item 8				0,774	
Item 14				0,593	
Item 17				0,697	
Item 4					0,635
Item 10					0,881
Item 15					0,747
Item 18					0,882
Variância média extraída	0,617	0,462	0,469	0,722	0,404
Variância total	0,38				

BREQ-2: *Behavioral Regulation in Exercise Questionnaire*.

n=118.

Os valores associados à variância média extraída dos fatores do modelo variaram de 0,469 (regulação externa) a 0,722 (regulação identificada). Os itens que mais discriminaram cada fator foram o 12 para amotivação (“Não percebo o objetivo de fazer exercício”); o 6 para regulação externa (“Participo no exercício porque meus amigos/família/parceiro dizem que devo fazer”); o 7 para regulação introjetada, (“Sinto-me envergonhado/a quando falto a uma sessão de exercício”); o 3 para regulação identificada (“Dou valor aos benefícios/vantagens do exercício”) e o 18 para regulação integrada (“Fico bem disposto e satisfeito por praticar exercício”).

### Estabilidade de dimensões nos formatos *Behavioral Regulation in Exercise Questionnaire*

A tabela 3 apresenta valores médios com respectivos desvios-padrão tanto para o formato tradicional impresso como para o *on-line* da aplicação do BREQ-2. O teste U de Mann-Whitney não mostrou diferença significativa nas dimensões do BREQ-2 entre os diferentes formatos. Houve também excelente consistência entre os formatos de administração em todas as dimensões, com valores de coeficiente de correlação intraclasse entre 0,78 (IC95%: 0,69-0,85) e 0,84 (IC95%: 0,77-0,89), sugerindo confiança desejável entre ambos os formatos de administração (Tabela 4).

**Tabela 3:** Perfis de associação entre as dimensões da versão *on-line* do *Behavioral Regulation in Exercise Questionnaire*

	Amotivação	Regulação externa	Regulação introjetada	Regulação identificada	Regulação integrada
Amotivação	1,00				
Regulação externa	0,74	1,00			
Regulação introjetada	0,15	0,45	1,00		
Regulação identificada	−0,15	0,06	0,73	1,00	
Regulação integrada	−0,34	−0,01	0,50	0,80	1,00

**Tabela 4:** Comparação e consistência entre as dimensões do *Behavioral Regulation in Exercise Questionnaire*

Dimensões do BREQ-2	Impresso	*On-line*	Valor de p*	CCI (IC95%)
	Média±DP	Média±DP		
Amotivação	0,51±0,79	0,43±0,72	0,170	0,83 (0,76-0,88)
Regulação externa	0,48±0,64	0,38±0,60	0,480	0,80 (0,71-0,86)
Regulação introjetada	1,56±1,05	1,33±1,10	1,000	0,78 (0,69-0,85)
Regulação identificada	2,59±0,81	2,43±0,92	0,120	0,79 (0,70-0,85)
Regulação integrada	2,45±1,05	2,24±1,12	0,100	0,84 (0,77-0,89)

* teste de Mann-Whitney.

BREQ-2: *Behavioral Regulation in Exercise Questionnaire*; DP: desvio-padrão; CCI: coeficiente de correlação intraclasse; IC95%: intervalo de confiança 95%.

## DISCUSSÃO

O presente estudo avaliou a validade e a consistência do BREQ-2 e forneceu evidências de sua aplicabilidade clínica em um contexto *on-line*. O modelo de cinco fatores do BREQ-2 *on-line* mostrou consistência interna, tanto em termos das dimensões da escala quanto do total de itens. De fato, todos os reguladores comportamentais extraídos apresentaram consistência interna, com alfa de Cronbach acima de 0,7 (exceto regulação externa, com alfa de 0,67).

Uma das explicações possíveis poderia estar na teoria por trás da regulação externa, que postula que as pessoas executam ações para satisfazer as expectativas daqueles ao seu redor. A regulação externa também pode ser interpretada como um sentimento de culpa ao faltar a uma sessão de exercício, o que torna esse fator semelhante à regulação introjetada.^(24)^ Entretanto, considerando que o fator associado à regulação externa apresenta um número maior de itens em comparação com a regulação introjetada, assume-se que a consistência interna poderia ser marginal.^(20)^ Esses achados sugerem que a administração *on-line* do BREQ-2 está associada a índices desejáveis de consistência interna.

Os achados deste estudo são semelhantes aos encontrados em outras versões do BREQ-2, traduzidas para outros idiomas. Quanto à estrutura fatorial do formato *on-line* do BREQ-2, os resultados apresentam níveis satisfatórios de ajuste, com disposição fatorial semelhante encontrada tanto no estudo original,^(15,16)^ como nas outras versões linguísticas.^(17,25-30)^ Medir a direção das associações entre as dimensões oferece outro método analítico para analisar a validade da estrutura fatorial teórica subjacente ao BREQ-2 *on-line*. Essas associações devem se mover na mesma direção que as respostas sobre o *continuum* da TAD. Neste caso, a covariância entre as dimensões apresentadas confirma a presença de um *continuum* da teoria de autodeterminação, pois as regulações próximas umas das outras no *continuum* se mostraram fortemente correlacionadas numa direção positiva, quando comparadas com regulações mais distantes no *continuum*.

Vários estudos demonstraram que a adaptação de questionários e de outros instrumentos de coleta de dados à Internet não compromete suas medidas.^(7-9,31)^ Relatórios de resultados mais positivos de instrumentos *on-line*, quando comparados com instrumentos impressos tradicionais, também foram descritos na literatura.^(9)^ É possível que a administração de questionários pela Internet reduza a tendência de fornecer respostas socialmente desejáveis. Isso porque os computadores poderiam proporcionar uma situação mais impessoal, na qual os indivíduos se sentem mais anônimos e menos preocupados com a forma como podem ser percebidos por outras pessoas.^(5,9)^

Não foram encontradas diferenças entre os dois formatos diferentes para administrar o BREQ-2 a estudantes de graduação. Foram observados valores elevados do coeficiente de correlação intraclasse, indicando excelente estabilidade nas respostas obtidas através do formato *on-line* do BREQ-2, em comparação com o formato tradicional impresso. Houve pequenas diferenças entre os formatos em termos de estrutura fatorial, quanto ao ajuste do modelo aos dados para administração *on-line*.

Apesar dos achados promissores, este estudo ainda apresentou várias limitações, que devem ser detalhadas. A primeira limitação, como em muitos questionários, é que todas as respostas são autorrelatadas. Isso significa que as respostas podem não corresponder necessariamente à atividade física real dos participantes. Entretanto, o autorrelato é um procedimento comum para coleta de dados usando questionários e outros instrumentos, pois é o método mais viável para coletar dados sobre percepções e crenças. O fato de certos itens *on-line* apresentarem pequenos desvios em relação a uma distribuição normal também deve ser lembrado. No entanto, neste estudo, a AFC foi realizada com pelo método de estimativa WLSMV com rotação oblíqua, que contempla a teoria subjacente e assume que os dados não têm distribuição normal multivariada.^(23)^ Outra limitação deste estudo consiste no fato de que, embora o tamanho da amostra fosse relativamente grande em comparação com o universo amostral, a seleção dos participantes não foi aleatória. Essa falta de seleção aleatória poderia significar que os participantes não eram verdadeiramente representativos da população mais ampla.

Não foram investigados aspectos socioeconômicos dos participantes, o que impediu avaliar a relação entre as respostas e esses dados da amostra, bem como generalizar os resultados. Porém, uma vez que o instrumento seja válido para aplicação *on-line*, outros estudos que avaliam a relação entre vários aspectos, incluindo as características sociodemográficas, podem ser realizados.

## CONCLUSÃO

As respostas obtidas pela administração do formato *on-line* do *Behavioral Regulation in Exercise Questionnaire* não foram estatisticamente diferentes dos valores obtidos com as respostas coletadas no formato impresso. Além disso, foram observados valores desejáveis no desempenho psicométrico, em relação à solução fatorial e às medidas de confiabilidade das respostas obtidas pela administração *on-line* do instrumento.
